# The effects of combined transcranial brain stimulation and a 4-week visuomotor stepping training on voluntary step initiation in persons with chronic stroke—a pilot study

**DOI:** 10.3389/fneur.2024.1286856

**Published:** 2024-02-21

**Authors:** Shih-Chiao Tseng, Dana Cherry, Mansoo Ko, Steven R. Fisher, Michael Furtado, Shuo-Hsiu Chang

**Affiliations:** ^1^Neuromechanics Laboratory, Department of Physical Therapy, University of Texas Medical Branch, Galveston, TX, United States; ^2^Department of Physical Therapy, University of North Texas Health Science Center at Fort Worth, Fort Worth, TX, United States; ^3^Neuromuscular Plasticity Laboratory, Department of Physical Medicine and Rehabilitation, University of Texas Health Science Center at Houston, Houston, TX, United States

**Keywords:** tDCS, motor learning, rehabilitation, gait initiation, anticipatory postural adjustment

## Abstract

**Purpose:**

Evidence suggests that transcranial direct current stimulation (tDCS) can enhance motor performance and learning of hand tasks in persons with chronic stroke (PCS). However, the effects of tDCS on the locomotor tasks in PCS are unclear. This pilot study aimed to: (1) determine aggregate effects of anodal tDCS combined with step training on improvements of the neural and biomechanical attributes of stepping initiation in a small cohort of persons with chronic stroke (PCS) over a 4-week training program; and (2) assess the feasibility and efficacy of this novel approach for improving voluntary stepping initiation in PCS.

**Methods:**

A total of 10 PCS were randomly assigned to one of two training groups, consisting of either 12 sessions of VST paired with a-tDCS (*n* = 6) or sham tDCS (s-tDCS, *n* = 4) over 4 weeks, with step initiation (SI) tests at pre-training, post-training, 1-week and 1-month follow-ups. Primary outcomes were: baseline vertical ground reaction force (B-vGRF), response time (RT) to initiate anticipatory postural adjustment (APA), and the retention of B-VGRF and RT.

**Results:**

a-tDCS paired with a 4-week VST program results in a significant increase in paretic weight loading at 1-week follow up. Furthermore, a-tDCS in combination with VST led to significantly greater retention of paretic BWB compared with the sham group at 1 week post-training.

**Clinical implications:**

The preliminary findings suggest a 4-week VST results in improved paretic limb weight bearing (WB) during SI in PCS. Furthermore, VST combined with a-tDCS may lead to better retention of gait improvements (NCT04437251) (https://classic.clinicaltrials.gov/ct2/show/NCT04437251).

## 1 Introduction

Persons with chronic stroke (PCS) often exhibit reduced body weight bearing (BWB) on the paretic leg, which further limits their ability to shift weight actively during activities of daily living such as standing, rising from sit-to-stand, and walking (1–4). The impaired ability of active weight shifting, coupled with restricted loading onto the paretic leg, leads to postural instability and asymmetrical gait patterns (5–8). Persistent gait deficits in PCS can also lead to a more sedentary lifestyle (9, 10). This sedentary behavior may elevate the risk of recurrent stroke and cardiovascular diseases (9, 11).

Gait initiation (GI) or step initiation (SI) is a useful paradigm for studying the control strategies of human locomotion from the perspective of the transition from upright stance posture to steady-state walking (12, 13). Prior to executing the first step, an automatic postural adjustment known as anticipatory postural adjustments (APA) is triggered in response to the destabilizing postural balance while standing, ensuring postural stability and a stable whole-body progression (12–14). Biomechanically, the APA consists of a series of postural adjustments. Initially, there is a backward shift of center of pressure (CoP) relative to the center of mass (CoM) position, which serves to break the static balance while standing. To quickly reestablish postural balance, there is an active weight shifting onto the stepping (or swing) leg, coupled with a simultaneous backward movement. This is followed by a rapid unloading of weight from the swing leg, shifting the body weight back to the standing leg (mediolateral CoP shift) for the step execution. The backward shift of CoP provides the initial propulsive forces to effectively progress the body forward and the active weight shifting helps disengage the stepping leg for step execution and is crucial for maintaining stability (12, 13, 15–17). Stroke commonly disrupts the automatic postural responses which contribute to standing balance (18–20). This can lead to difficulty walking and increased risk of falling (21–26).

An important motor learning and control principle underlying post-stroke gait rehabilitation is task-specific training. Stepping training practice is one of the locomotor tasks commonly implemented in standard clinical care as well as research studies for post-stroke gait intervention (1, 27, 28). We have developed a visuomotor stepping task (VST) which uses real-time, augmented visual feedback (VF) to enhance the individual’s kinesthetic sense of the paretic leg for improving forward stepping control (29). VST uses real-time visual feedback to guide the paretic leg stepping forward onto a specific visual target. Additionally, at the end of each stepping trial, visual feedback regarding the final stepping foot position in relation to the visual target location (i.e., movement error) is provided to increase the individual’s awareness of stepping control deficits and acts as a powerful motivator to improve stepping accuracy by reducing the movement errors on a trial-by-trial basis.

Transcranial direct current stimulation (tDCS) is a non-invasive, low-intensity brain stimulation technique to modulate neural excitability and enhance motor performance and learning of motor tasks in humans (30–34). Depending on the stimulation polarity, tDCS can upregulate or downregulate cortical excitability, facilitating or impeding skill performance and learning (35–38). tDCS can enhance motor learning and recovery of arm and hand functions in healthy and stroke populations (30, 33, 39–48). However, the additive effects of tDCS on lower limb motor recovery and gait improvements in PCS remain inconclusive (49–54). For example, Groin et al. investigated the additive effects of anodal tDCS (a-tDCS) on robot-assisted gait training in PCS and showed there were no differences in six-minute walking and 10-meter walking performances between a-tDCS and sham tDCS (s-tDCS) groups after robot-assisted gait training combined with anodal/sham tDCS (53). In contrast, another study showed that a-tDCS combined with conventional physical therapy enhanced the recovery of lower extremity motor function post stroke (49). Specifically, the a-tDCS group had greater improvements in Fugl-Meyer Lower Extremity (F-M LE) and lower limb Motricity Index (MI-LE) compared to the sham group; However, there were no significant differences between the anodal and sham groups for Berg balance Score and gait performance post training (49). More studies are needed to develop the optimal dose–response relationship between brain stimulation and gait training for PCS.

Taken together, PCS may benefit from a new training paradigm that pairs VST training with a-tDCS. Real-time visual feedback can be used to improve paretic stepping control while a-tDCS can enhance increase cortical excitation and thereby enhance learning of motor tasks (1, 27, 28, 30–34). While we demonstrated that a-tDCS in conjunction with a visuomotor stepping task (VST) can enhance retention of the stepping skill for 30 min post-stimulation in healthy adults (29), no studies have examined the combined effects of a-tDCS with VST on the improvements of APA and weight shifting on the paretic leg during SI. Thus, the purpose of this pilot study was to investigate the effects of a 4-week VST on the improvements of APA and symmetry of limb loading in PCS, and (2) to determine the additive effects of a-tDCS when paired with VST on the improvements and retention of symmetry of limb loading in PCS. We hypothesized that the a-tDCS group would show greater improvements and longer retention of ground walking compared with the sham-tDCS (s-tDCS) group. A better understanding of whether a-tDCS enhances the ability to retain gait training improvements post stroke could guide the development of effective intervention strategies for stroke gait rehabilitation.

## 2 Methods

### 2.1 Participants

Ten individuals with a history of a single cerebrovascular accident who met the inclusion criteria were enrolled in this pilot study, but three participant’s pre-training data were unable be analyzed due to data loss caused by a hard drive crashed. The clinical and demographic characteristics of the participants are summarized in Table 1. The inclusion criteria were: (1) age of 18 to 85 years, (2) a history of unilateral, first-ever ischemic stroke more than 6 months before study enrollment, (3) presence of residual gait deficits (i.e., visible gait asymmetry) but ability to walk with/without assistive devices continuously for 5 min at self-selected speed, (4) Mini-Mental State Examination (MMSE) score > 21 in order to follow instructions. The exclusion criteria were: (1) existing medical conditions that restrict exercise training, (2) presence of malignant neoplasm or tumors, (3) receptive or global aphasia, and (4) contraindications to brain stimulation, including pregnancy, history of seizures, any metal implants, cardiac pacemakers, or use of medications that alter cortical excitability. Participants gave informed consent prior to participation and the study was approved by the Texas Woman’s University Institutional Review Board.

**Table 1:** Subject characteristics.

Group	Age	Gender	Weight (Kg)	Height (cm)	Paretic leg	Duration of injury (years)	MMSE	F-M
**a-tDCS**								
1	62	F	86	180	R	8	27	26
2	70	M	123	188	R	5	26	25
3	40	M	82	185	R	2	29	14
4	59	F	45	152	L	8	30	28
5	58	M	75	180	L	5	30	27
6	56	F	68	160	L	4	30	22
Mean	57.50	3F, 3 M	79.83	174.17		5.33	28.67	23.67
SD	9.87		25.62	14.62		2.34	1.75	5.16
**s-tDCS**								
1	30	M	95	185	L	12	30	34
2	49	F	68	165	L	9	30	31
3	45	F	54	160	L	2	28	23
4	44	M	85	180	R	3	29	18
Mean	42.00	2F, 2 M	75.50	172.50		6.50	29.25	26.50
SD	8.29		18.16	11.90		4.80	0.96	7.33

a-tDCS, anodal transcranial direct current stimulation; s-tDCS, sham transcranial direct current stimulation. MMSE, mini-mental state examination; F-M, Fugl-Meyer Lower Extremity score.

### 2.2 Experimental design

**Figure 1:**
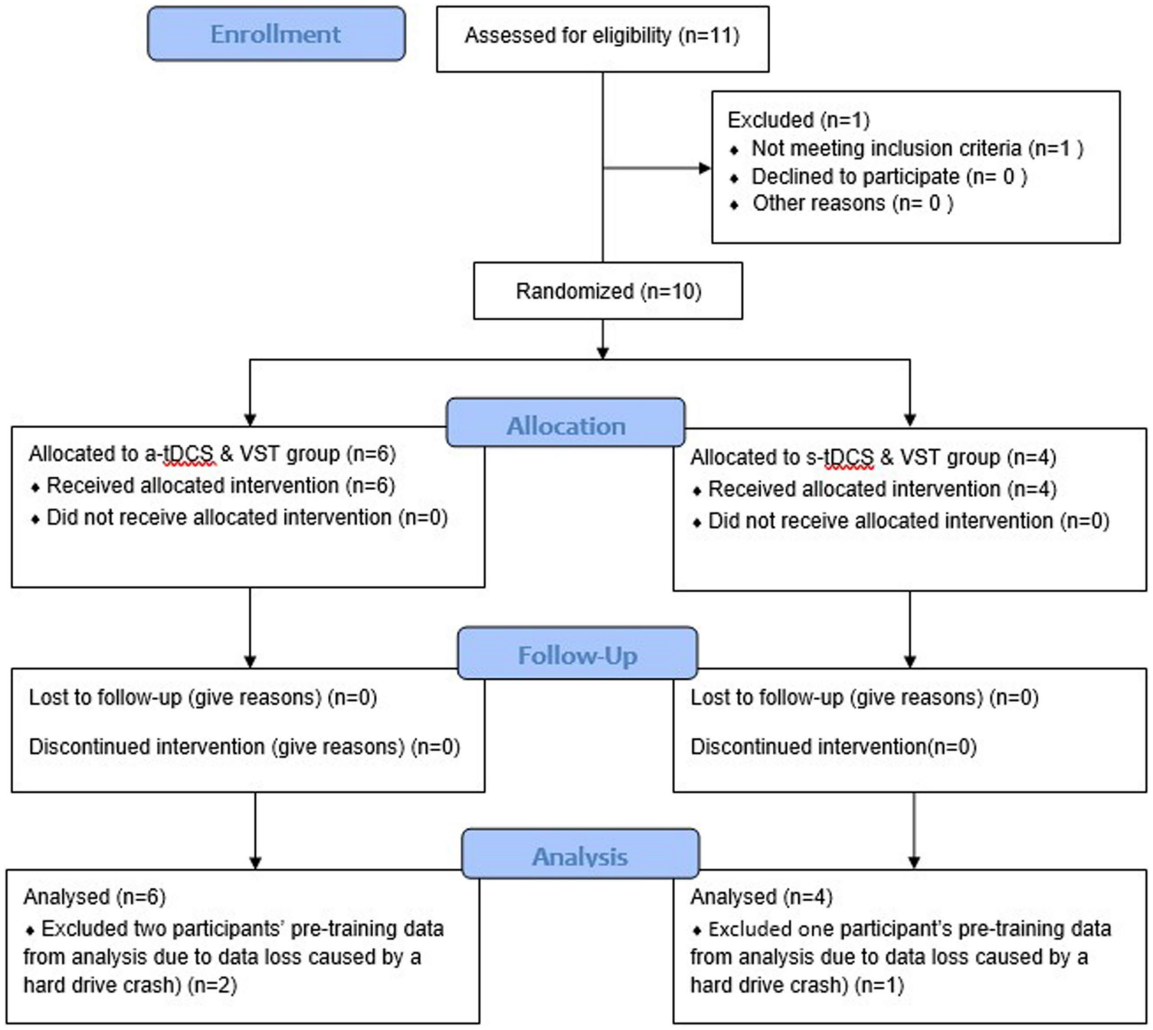
Consolidated Standards of Reporting Trials (CONSORT) Flow Chart. A total of 10 participants were enrolled and randomly assigned into one of two intervention groups: anodal transcranial direct current stimulation (a-tDCS) or sham transcranial direct current stimulation (s-tDCS) groups. All participants completed a four-week visuomotor stepping training (VST) paired with the assigned tDCS and two follow-up tests after the completion of the intervention (one-week and one month follow-up tests). Data from ten participants were analyzed, including data loss of three participants’ pre-training data caused by a hard drive crash.

**Figure 2:**
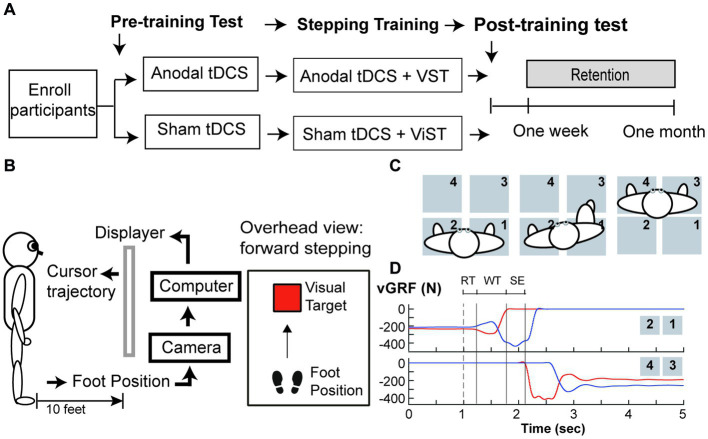
Study Protocol (A) Study Design, (B) Set up for visuomotor stepping task (VST), (C) Set up for the ground reaction forces (GRF) measured during step initiation, and (D) Vertical ground reaction force (vGRF) recorded by four force plates during a step initiation trial. (A) Participants were randomly assigned to one of two tDCS groups (anodal or sham tDCS) and underwent twelve training sessions over a four-week period. GRF were measured before and one day after training, one week and one month after training. (B) Subjects learned to move the foot cursor to a visual target using real-time visual feedback and were instructed to step onto the target as fast as possible and as accurate as possible without loss of the balance. This stepping training were paired with either either anodal or sham tDCS. (C) An overhead view of the experimental setup for GRF measures under the paretic leg and non-paretic leg during step initiation. In a step trial, the participant was instructed to take a forward step using his paretic leg onto the force plates (#3) in front of him, followed by taking another step forward using his non-paretic leg(#4). (D) An illustration of vGRF exerted under the paretic (Red lines) and the non-paretic leg (Blue lines) from four force plates during a step initiation trial. A cutaneous electrical stimulation (i.e. dash line) was used to cue the participant to make a voluntary step forward. The onset of the anticipatory postural adjustment (APA) was determined by first increasing vGRF under the paretic leg (i.e. upper panel, red line). The vGRF data under the stepping leg were used to determine the duration of three movement phases: response time (RT), weight transfer (WT), and step execution (SE) phases.

This pilot study was a randomized-controlled, placebo-controlled, and single-blind, intervention study. This study was a part of a larger research project that included a cross-sectional study (Phase I) followed by an exploratory, intervention study (Phase II). The researchers were not aware of trial registration during Phase I study; the study was later registered in ClinicalTrial.gov (NCT04437251) prior to the first subject enrollment for Phase II study (Figure 1). Following enrollment, participants were randomly assigned to one of two groups (a-tDCS or sham tDCS; s-tDCS) based on a random series of binary numbers that was generated by a computer program (MatLAB, MathWorks Inc.) prior to the enrollment (Figure 2A). All participants then underwent a total of 12 training sessions over 4 weeks during which participants learned a novel visuomotor stepping task (VST) while 20-min of tDCS (a-tDCS or s-tDCS) was delivered over the leg area of the M1 (29, 54). The designated trainers administered the tDCS protocol to the participants according to their group assignments in each training session. However, all participants were blinded to the stimulation protocol. SI was measured before and 1 day after the 4-week stepping training. SI was then measured 1 week and 1-month post-training to examine the effects of tDCS on the retention of locomotor skill training. Three designated trainers were involved in daily exercise training, but they were not involved in any functional or laboratory measures. Additional one assessor who was not involved in the exercise training was designated to conduct all tests; to minimize the measurement bias, the assessor had followed the standardized testing procedures, including the pre-written script for verbal instructions and the standardized experimental protocol of force plate setup for force measures. The assessor administered the same tests to all participants regardless of their group assignments.

#### 2.2.1 VST

A camera system (OptiTrack Inc.) and Custom C++ program were used to record the real-time foot trajectory at a sampling rate of 100 Hz, and to create real-time visual animation displayed via a projector on the wall screen during stepping training (29). A reflective marker was attached to the stepping foot (i.e., either paretic or non-paretic foot) to indicate the real-time cursor’s location, and participants were given real-time visual feedback about their leg movements via a foot cursor displayed on the wall screen 3 meters (10 feet) away from the front view (Figure 2B). The task was to move the cursor from a start location to the target at a safe and fast speed. In each trial, the target (red square) was programmed to appear at a random time between 1 and 3 s after the trial was started. Participants were instructed to make two consecutive forward steps at the fastest speed, starting with the stepping foot followed by the trailing foot. The goal was to move the virtual cursor into a visual target as soon and as accurately as possible. At the end of each trial, participants were instructed to take two consecutive backward steps to return to the starting position. Thus, to complete a VST in each trial, participants were required to make two forward steps and two backward steps. Each training session took about 1 h and comprised 140 trials, including 40 trials before brain stimulation (20 trials/leg), 80 trials during tDCS brain stimulation (40 trials/leg), and 20 trials immediately after brain stimulation (10 trials/leg). Thus, in each training session, participants were trained to make 280 forward visually guided steps and 280 backward steps to resume the initial location.

#### 2.2.2 tDCS protocols

Twenty minutes of brain stimulation (either s-tDCS or a-tDCS) was delivered through a pair of saline-soaked sponge electrodes after participants completed the first 40 trials in each session (29). The anodal or sham electrode was placed over the leg area of the lesioned motor cortex and the reference electrode was placed over the contralateral supraorbital ridge. For the a-tDCS (i.e., real brain stimulation) group, the stimulus intensity was set to 2 mA over a 20-min period. For the s-tDCS (i.e., placebo stimulation) group, 2 mA current was delivered in the first 10 s and was turned off for the remainder of the 20-min period. Therefore, the participants in both stimulation groups felt a subtle tingling sensation in the first 20 s when the current was gradually increasing to 2 mA because the current intensity was very low. They became accustomed to the stimulation and no longer felt it throughout the rest of the session. This sensory adaptation meant that participants could not differentiate one stimulation protocol from the other (i.e., active vs. sham tDCS), and thereby blinding of the participants to the treatment assignments (29). During training, the trainers closely monitored the participant’s response to tDCS and the participants were asked any pain or discomfort due to the stimulation. tDCS would be immediately terminated if any pain or discomfort was reported by the participants.

### 2.3 Data collection

#### 2.3.1 Baseline cognitive and lower extremity motor function assessments

Before starting the training, the Mini-Mental State Examination (MMSE) and Fugl-Meyer LE motor test (F-M) were used to assess participants’ cognitive and LE motor functions (55, 56).

#### 2.3.2 Ground reaction force

Four force plates (AMTI Inc.) were used to measure force exerted under the paretic and non-paretic legs during a voluntary SI (see Figure 2C). Ground reaction force (GRF) data were collected at 1,000 Hz. The participants maintained a quiet standing posture with each leg placing on two separate force plates. They were instructed to make a forward step onto the initiate as fast as possible once they sensed a small cutaneous electrical stimulation delivered to the popliteal fossa of the paretic leg. Three step trials were collected during pre-training, post-training, 1-week, and 1-month follow-up tests.

### 2.4 Data analyses

Force data and time events were calculated using custom MATLAB (Mathworks, Natick, MA) software. Offline, force data were filtered by a low-pass Butterworth filter with a cut-off frequency set at 10 Hz. Three time events were identified based on the changes in vertical GRF (vGRF) under the paretic and non-paretic leg: (1) paretic heel-off (H-Off; vGRF > = 20 N), (2) paretic heel-on (H-On; vGRF > = 20 N), and (3) the onset of APA (APA-On; vGRF under the non-paretic leg first decreased more than 5% of its baseline mean value during quiet standing) (26, 57). The durations of the three movement phases were calculated based on these events within each step trial (see Figure 2D): response time (RTP), weight transfer (WTP), and stepping execution (SEP) phases (26, 57); RTP was the time duration from the electrical stimulation to APA-on; WTP was the time duration from the end of RTP to onset of heel-off on the stepping leg. The WTP encompasses the very well-characterized and stereotyped lateral weight shift, first toward the stepping side, then the supporting side, that rapidly unloads the stepping leg before SI. SEP was the time duration from the end of WTP to subsequent heel strike of the stepping leg onto the target. Three force variables were calculated to determined changes in WB and force production: (1) percent of BWB under the paretic leg during one-second quiet standing period (i.e., baseline) prior to the electrical stimulation (B-vGRF) and (2) peak vGRF developed under the paretic leg prior to SI (P-vGRF). All force data were normalized to individual body weight (N/Kg). The B-VGRF was then expressed as the percent of the sum of paretic and non-paretic vGRF.

To quantify the effects of tDCS on the skill retention of the paretic BWB and onset of APA during SI, we calculated the retention of paretic B-vGRF and RTP, relative to the post-training values, normalized to its mean values measured 1 day post-training (29, 58). This allowed us to account for differences among individuals, thereby comparing changes in paretic BWB and APA in the real world (i.e., home and community) after completion of the 4-week stepping training. A value of 100% indicated no change in paretic BWB at 1 week or 1 month post-training. Decreasing this percentage therefore reflects less retention of the training effect over time. Group means were calculated for two follow-up time points (1-week and 1-month post-training).

### 2.5 Statistics

Statistical comparisons between two groups (a-tDCS vs. s-tDCS) across four time points (pre- and post-training, 1-week and 1-month follow-up tests) were made using SAS software (SAS Inc., Cary, NC). A two-way mixed model analysis of variance (ANOVA) with repeated measures (groups × testing times) was used to compare differences in group reaction forces and movement phases between groups and across four testing times to quantify weight transfer and BWB improvement on the paretic leg associated with stepping training and brain stimulation. When ANOVA was significant, post-hoc analyses were performed using Tukey’s honestly significant difference (HSD) test. The level for statistical significance was set at *p* ≤ 0.05.

## 3 Results

Figure 2D illustrates the plots of vGRF exerted under the paretic (red lines) and non-paretic leg (blue lines) from a representative participant during SI. The paretic vGRF was increased during APA prior to paretic heel-off (Figure 2D, upper, red line). Table 2 provides statistical comparisons between groups at four time points for movement phases and GRF measures. The a-tDCS group significantly increased paretic B-vGRF from their pre-training and post-training values at 1 week post-training (*post hoc*, *p* = 0.007 and 0.0007, respectively). After normalization to the post-training B-vGRF, the retention of paretic B-vGRF was significantly higher in the a-tDCS group compared with the s-tDCS group (main effect of group, *p* = 0.036). There was a significant interaction effect for the retention of paretic B-vGRF (*p* = 0.02). The retention of paretic B-vGRF 1-week post-training was significantly higher in the a-tDCS group compared with the s-tDCS group (*post hoc*, *p* = 0.008), suggesting that a-tDCS may help retain the training-induced BWB improvement on the paretic leg up to 7 days post-training. Additionally, the a-tDCS group showed a significant increase in retaining paretic B-vGRF at 1 week post-training compared with its post-training average (*post hoc*, *p* = 0.003).

**Table 2:** Outcome variables of the step initiation test (means ± standard deviations).

	a-tDCS	s-tDCS	Group	Time	Group × time interaction
Response Time Phase (RTP, millisecond)			0.67	0.17	0.83
Pre-training	565.73 ± 433.18	514.75 ± 128.34			
Post-training	397.89 ± 292.75	311.25 ± 203.68			
One-week	288.45 ± 142.20	305.83 ± 233.56			
One-month	519.93 ± 456.09	405.92 ± 116.33			
Weight Transfer Phase (WTP, millisecond)			0.18	0.31	0.54
Pre-training	852.33 ± 523.86	676.25 ± 191.27			
Post-training	990.67 ± 333.43	762.58 ± 254.65			
One-week	1639.50 ± 1045.09	848.50 ± 314.28			
One-month	983.80 ± 456.30	859.67 ± 396.67			
Step Execution Phase (WTP, millisecond)			0.22	0.68	0.34
Pre-training	714.93 ± 253.63	531.25 ± 79.55			
Post-training	888.47 ± 570.28	467.96 ± 98.25			
One-week	604.94 ± 285.78	537.09 ± 120.16			
One-month	586.34 ± 243.51	500.67 ± 88.75			
^*^Baseline Paretic Weight Bearing (B-vGRF, %)			0.82	0.03	0.009
Pre-training	39.46 ± 6.85	44.59 ± 11.34			
Post-training	41.94 ± 14.77	45.57 ± 7.07			
One-week	53.63 ± 11.68	30.7 ± 5.02		1-wk > Post-T, *p* = 0.035	a-tDCS, 1-wk > Pre-T, *p* = 0.007
					a-tDCS, 1-wk > Post-T, *p* = 0.0007
One-month	48.51 ± 14.78	25.5 ± 3.39			
Peak Paretic Weight Bearing (P-vGRF, N/Kg)			0.77	0.13	0.08
	−5.21 ± 1.15	−5.49 ± 1.34			
	−4.37 ± 3.09	−5.96 ± 1.55			
	−6.73 ± 1.88	−5.82 ± 2.29			
	−5.99 ± 2.07	−6.25 ± 1.57			
Retention of RTP (%)			0.26	0.08	0.53
Post-training	0	0			
One-week	−14.38 ± 41.58	4.35 ± 52.38			
One-month	18.59 ± 46.22	69.07 ± 95.83			
^**^Retention of B-vGRF (%)			0.036	0.06	0.02
Post-training	100	100			
One-week	132.89 ± 25.37	96.08 ± 11.50	a-tDCS > s-tDCS		1-wk: a-tDCS > s-tDCS; *p* = 0.008
					a-tDCS: 1-wk > Post-T; *p* = 0.003
One-month	115.03 ± 13.14	101.43 ± 12.33			

^*^Significant effects of time and interaction of group by time. ^**^Significant effects of group, and interaction of group by time. P, paretic; Post-T, post-training; Pre-T, pre-training; 1-wk, one-week follow-up test; 1-mon, one-month follow-up test; B-vGRF, baseline vertical ground reaction force; P-vGRF, peak vertical ground reaction force.

There were no adverse effects (i.e., pain or discomfort) reported by the participants in this study. Although not formally documented in the training logs, approximately 50% of the participants in each group have reported the most common side effects (i.e., mild itching or tingling) during current ramp up (i.e., the first 20 s of stimulation) and this sensation started to fade within the first 2 mins of the stimulation; afterward, they no longer feel anything throughout the training session. These symptoms were mild and transient and did not affect their performance in each training session.

## 4 Discussion

In this pilot study, we investigated the combined effect of a-tDCS and VST training on the improvement of weight transfer and loading on the paretic leg during SI in PCS, and its retention over a one-month follow-up period. The study showed that a-tDCS paired with a 1-week VST program results in a significant increase in paretic weight loading at 1-week follow up. Furthermore, a-tDCS in combination with VST led to significantly greater retention of paretic BWB compared with the sham group at 1 week post-training.

### 4.1 a-tDCS combined with VST leads to significantly greater retention of paretic BWB 1-week post-training

The primary finding was that a-tDCS improved the retention of paretic BWB for PCS up to 7 days after completion of a 4-week VST program. The paretic BWB in the a-tDCS group was significantly increased at 1-week follow-up compared to its pre-training and post-training values, suggesting a-tDCS may help with short-term retention of improved paretic limb loading. tDCS has been used as an adjuvant to motor skill learning and therapeutic interventions (or functional training) for enhancing learning and motor functions in health and disease. A general assumption is that the application of tDCS enhances motor cortical excitability, leading to improved motor performance and motor learning (59). Furthermore, the increased neural excitability persists even after cessation of stimulation, referred to as a long-lasting “after-effect” (60, 61). In humans, anodal tDCS can produce a persistent after-effect, which increased neural excitation to up to 150% of its baseline value; this excitatory effect can last for approximately 60–90 min after the end of stimulation (54, 61, 62). It has been postulated that this long-lasting neural excitation may promote better skill retention after training (e.g., the retention of the paretic body weight bearing) (59). Thus, over the course of motor skill acquisition/training phases, the effects of a-tDCS-induced skill improvement (“skill gains”) can take place during the practice session (online learning) and between training sessions (offline learning). After completion of training, a-tDCS may help maintain the skill gains (“skill retention”) in the absence of skill practice or training (33, 59). In this pilot, we aimed to investigate the immediate and short-term impacts of a-tDCS on paretic BWB in PCS at three time points: 1 day (skill gains primarily via online learning), 1 week and 1 month post training (skill retention via consolidation). The preliminary results suggest that both a-tDCS and s-tDCS groups showed similar degrees of paretic BWB improvement immediately after a 4-week VST program. The finding is consistent with previous studies suggesting that tDCS-induced learning enhancement is task-specific (31, 50). In other words, tDCS augments motor skill learning restricted to the specific “trained” skill (stepping task) and can augment other locomotor skill performances (e.g., SI or GI). A study of hand pinch task learning in PCS showed that, when tDCS was added to a five-day training, hand pinch performance was significantly better relative to sham, mostly in online learning (i.e., during practice sessions) although additive effects of tDCS did not generalize to other “untrained” hand skills (e.g., Jebsen Taylor hand function test). The effect of this hand pinch task led to a significant increase in isometric pinch force on the paretic hand (50). Our pilot study suggested that a-tDCS may enhance short-term paretic BWB maintenance up to 7 days after the termination of stepping training. Compared with motor training alone (i.e., VST with s-tDCS), in the absence of skill practice, the a-tDCS group had a greater degree of skill retention for paretic BWB from its post-training values at 1 week follow-up; the s-tDCS group had a significant lower paretic BWB retention at 1-week follow-up compared to the a-tDCS group. Findings suggest that for PCS, a-tDCS enhances the short-term retention of paretic BWB improvements primarily via the offline effects – likely through consolidation. This short-term, a-tDCS-induced enhancement of skill retention has important scientific and practical implications for stroke rehabilitation.

### 4.2 a-tDCS does not improve APA during SI in PCS

The preliminary results suggest that there was no additive effect of a-tDCS on the improvements of APA during SI in PCS. Both a-tDCS and s-tDCS groups showed similar degrees of the RTP improvements immediately after a 4-week VST program. The RTPs post training for both a-tDCS and s-tDCS groups (397.89 and 311.25 ms respectively) were not different from their pre-training values (565.73 and 514.75 ms respectively). During GI or SI, PCS often presents impaired APA compared to healthy adults, characterized by abnormal APA patterns (no APA or multiple APAs), impaired muscle activations, slower APA initiation, longer APA duration, and lower GRF amplitudes (20, 21, 63, 64). Consistent to previous studies in healthy adults and PCS, both a-tDCS and s-tDCS groups demonstrated impaired APA, characterized by slower RTPs (i.e., delayed initiation of APA) and longer WTPs (i.e., prolonged APA duration). A slower RTP and prolonged APA duration suggest a decreased ability to initiate and organize APA effectively, essential for maintaining postural stability and forward body progression prior to initiating a forward step. Furthermore, impaired APA in PCS has negative impacts on gait performance as research has shown that impaired APA is correlated with slower gait speed, shorter step length, greater motor impairment, less motor recovery, and poor balance (7, 20, 63).

### 4.3 VST is feasible to be an effective intervention to facilitate APA during GI in PCS

Decreasing RTP after a 4-week VST program in PCS has important implications for stroke gait rehabilitation as VST can be used to target PCS who has impaired or no APA during GI. In this pilot study, each participant had completed a total of 1,680 stepping training trials within 4 weeks, including 840 trials with the use of the paretic leg as the leading leg and 840 trials with the use of the non-paretic leg as the leading leg during SI. Research has shown that decreased paretic tibialis anterior (TA) muscle activation is the main mechanism underlying impaired or absence of APA during GI in PCS (5, 8, 12, 18, 65). Furthermore, it has been reported that the paretic TA activation was increased by between 27% and 36% when initiating gait with the nonparetic limb in PCS (8). Therefore, the main mechanism underlying the improvement of RTP after a 4-week VST program is likely associated with the practice of using the nonparetic leg as a leading leg during stepping training, leading to improving paretic TA activation and APA during SI. Increasing paretic TA muscle activity during SI or GI serves to provide initial propulsive forces that shift the whole-body forward (5). This can lead to improving postural stability and propulsive force production; therefore, translate to a significant improvement in gait performances for PCS.

The results from this pilot study also suggest that VST combined with a-tDCS may enhance the excitation of the lesioned primary motor cortex (M1), leading to improvements of paretic locomotor control for PCS. It is known that motor skill learning is accompanied by a transient increase in corticospinal excitability as quantified by increased motor evoked potential (MEP) amplitudes after training (66–69). Furthermore, motor skill training accompanied by motor priming strategies such as noninvasive brain stimulation (i.e., tDCS) can increase M1 excitation and enhance skill learning. The premise is that tDCS-induced increases in cortical excitation will be further augmented when accompanied by learning a goal-directed task (i.e., VST). For stroke gait rehabilitation, a relatively new motor priming strategy—a combination of tDCS and motor skill training—has been proposed for maximum motor priming benefit as both modalities (i.e., skill training and tDCS) result in increased corticospinal excitation (58, 70–74). The findings from this pilot study suggest that VST combined with a-tDCS may increase cortical excitation of the lesioned M1which in turns improves paretic weight bearing during gait initiation in PCS.

tDCS appears to be safe and feasible for inpatient and outpatient rehabilitation settings as well as home-based applications (75–78). The safety, technical parameters, and application guidelines of tDCS have been investigated and recommended to minimize adverse effects and ensure safety of the participants in research (78–81). A recent systematic review from 18,000 research sessions in about 8,000 subjects found no evidence of serious adverse events or neuronal damages in a variety of populations, including the stroke population (79). The most common side effects include itching (a-tDCS vs. s-tDCS group: 39.3% vs. 32.9%), tingling (22.2% vs. 18.3%), headache (14.8% vs. 16.2%), burning sensations (8.7% vs. 10%) and discomfort (10.4% vs. 13.4%), with no significant differences between active and control groups (78, 82). Consistent with previous studies in healthy adults and PCS, the participants in this study reported the most common side effects including itching or tingling during current ramp up or ramp down (78, 83, 84). These symptoms were mild and transient and did not affect their performance in each training session, Overall, the findings from this pilot study consistent with previous research supporting the safety of tDCS (79, 83).

Taken together, the findings from this pilot study suggest that VST may be feasible for improving the paretic BWB, leading to improving postural stability during GI and overall gait performances in PCS. Moreover, a-tDCS paired with stepping training can Improve the short-term retention of paretic BWB post stepping training.

### 4.4 Limitations

There are two major limitations in this study that could be addressed in future research, The first limitation is the generalization of these results to the chronic stroke population due to small sample size. The second limitation concerns the baseline group differences which may result in different capabilities for GRF productions during SI. The participants in the s-tDCS group are younger with higher baseline lower-extremity motor function and may have a greater potential for GRF improvements following a 4-week VST compared to those in the a-tDCS group. This pilot study took the first step to explore the feasibility of combing stepping training and tDCS on the retention of paretic BWB and weight transfer in PCS. Future research studies with larger samples or clinical trials are needed to gain insights of neurological mechanisms underlying tDCS on the APA improvement and paretic BWB retention in PCS.

### 4.5 Clinical implications

Findings from this pilot study may have important clinical implications for geriatric population and individuals with neurological disorders, who have decreased ability to initiate and organize APA during GI, leading to postural instability and poor gait performance.

## Data availability statement

The original contributions presented in the study are included in the article/supplementary material, further inquiries can be directed to the corresponding author.

## Ethics statement

The studies involving humans were approved by Texas Woman’s University Houston IRB. The studies were conducted in accordance with the local legislation and institutional requirements. The participants provided their written informed consent to participate in this study.

## Author contributions

SC-T: Conceptualization, Formal Analysis, Methodology, Writing – original draft, Writing – review & editing, Funding acquisition, Investigation, Supervision, Validation. DC: Formal Analysis, Writing – review & editing. MK: Writing – review & editing. SF: Writing – review & editing. MF: Writing – review & editing. S-HC: Writing – review & editing, Conceptualization, Investigation, Methodology, Writing – original draft, Supervision.
